# Quantification of breast biopsy clip marker artifact on routine breast MRI sequences: a phantom study

**DOI:** 10.1186/s41747-024-00525-2

**Published:** 2024-11-15

**Authors:** Christian Kremser, Leonhard Gruber, Matthias Dietzel, Birgit Amort, Wolfram Santner, Martin Daniaux

**Affiliations:** 1 Department of Radiology, Medical University Innsbruck, Innsbruck, Austria; 2 Department of Radiology, University Hospital Erlangen, Erlangen, Germany; 3 Thurmed AG, Radiologie Südost, Chur, Switzerland

**Keywords:** Artifacts, Biopsy, Breast neoplasms, Clips, Magnetic resonance imaging

## Abstract

**Background:**

To investigate the artifact sizes of four common breast clip-markers on a standard breast magnetic resonance imaging (MRI) protocol in an *in vitro* phantom model.

**Methods:**

Using 1.5-T and 3-T whole-body scanners with an 18-channel breast coil, artifact dimensions of four breast biopsy markers in an agarose-gel phantom were measured by two readers on images obtained with the following sequences: T2-weighted fast spin-echo short inversion time fat-suppressed inversion-recovery with magnitude reconstruction (T2-TIRM); T1-weighted spoiled gradient-echo with fat suppression (T1_FL3D), routinely used for dynamic contrast-enhanced imaging; diffusion-weighted imaging (DWI), including a readout segmented echo-planar imaging (RESOLVE-DWI) and echo-planar imaging sequence (EPI-DWI). After outlining the artifacts by freehand regions of interest, sagittal and lateral diameters in axial images were measured.

**Results:**

Interreader agreement for artifact size quantification was high, depending on the sequence (80.4–94.8%). Overall, the size, shape, and appearance of artifacts depended on clip type and MRI sequence. The artifact size ranged from 5.7 × 8.5 mm^2^ to 13.4 × 17.7 mm^2^ at 1.5 T and from 6.6 × 8.2 mm^2^ to 17.7 × 20.7 mm^2^ at 3 T. Clip artifacts were largest on EPI-DWI and RESOLVE-DWI (*p* ≤ 0.016). In three out of four clips, T2-TIRM showed the smallest artifact (*p* ≤ 0.002), while in one clip the artifact was smallest on T1_FL3D (*p* = 0.026). With the exception of one clip in the RESOLVE sequence, all clips showed a decrease in the artifact area from DWI to ADC images (*p* ≤ 0.037).

**Conclusion:**

Breast clip-marker MRI artifact appearances depend on clip type, field strength, and sequence and may reach a significant size, potentially obscuring smaller lesions and hindering accurate assessment of breast tumors.

**Relevance statement:**

Considerable variations in artifact size and characteristics across different breast clips, MRI sequences, and field strengths exist. Awareness of these artifacts and their characteristics is essential to ensure accurate interpretation of scans and appropriate treatment planning.

**Key Points:**

- Awareness of breast clip artifacts is essential for accurate interpretation of MRI.
- The appearance of artifacts depends on breast clip type, field strength, and sequence.
- Clip-related artifacts might hinder the visibility of small lesions.

**Graphical Abstract:**

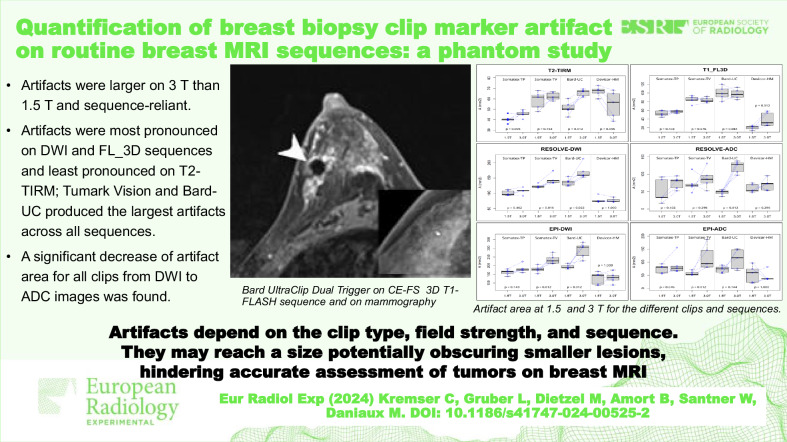

## Background

Breast cancer is one of the leading causes of cancer death among women in industrialized countries [1]. To ensure successful treatment early detection and diagnosis are important, which conventionally is performed using x-ray mammography and sonography. Due to the limited sensitivity and specificity of these methods particularly in patients with dense breast parenchyma or postsurgical scars, breast magnetic resonance imaging (MRI) has become a valuable tool in the management of selected breast cancer patients.

MRI is recognized as being very sensitive for the detection of primary or recurrent breast cancer and for the evaluation of response to therapy [2–4]. It even has been suggested by previous studies that MRI is superior to the other modalities in evaluating the size and extent of disease [5]. For patients with non-metastatic breast cancer local therapy usually is done by surgical resection with eventual postoperative radiation and increasingly systemic therapy before surgery [6]. During preoperative chemotherapy, some tumors show substantial clinical response. In this case, the tumor may no longer be palpable or visible on mammography or sonography at the time of surgery. This led to the use of clips to mark the tumor bed and thus allow identification of the lesion location at the time of surgery [7–9]. It has been suggested that the placement of tumor-marker clips should be an integral part of the multidisciplinary approach in appropriate patients [10] and has become standard practice.

At our institution, marker clips are routinely placed after ultrasound-guided biopsies, as a reference for follow-up or excision, as suggested [9]. During MRI follow-up or local staging prior to treatment, these maker clips, which are typically made from non-ferromagnetic metals, will result in image artifacts being noticeable as signal alterations and distortions [11] and may lead to misinterpretations or the inability to detect the respective lesion.

Current state-of-the-art breast MRI protocols follow a multiparametric approach consisting of T1-weighted contrast-enhanced imaging, T2-weighted, and diffusion-weighted imaging (DWI) [12, 13]. To our knowledge, scientific evidence on whether this leads to relevant image artifacts in breast MRI is very limited [11]. The purpose of this *in vitro* study was to quantify the extent of artifacts of four different breast clip markers commonly used in our clinical practice in a standard breast imaging MRI protocol.

## Methods

### Study type and ethics committee vote

The presented data stem from an experimental *in vitro* study. An ethics committee approval was granted for the use of comparative *in vivo* patient images (vote number 1085/2023).

### Phantom and breast biopsy clip marker information

**Fig. 1:**
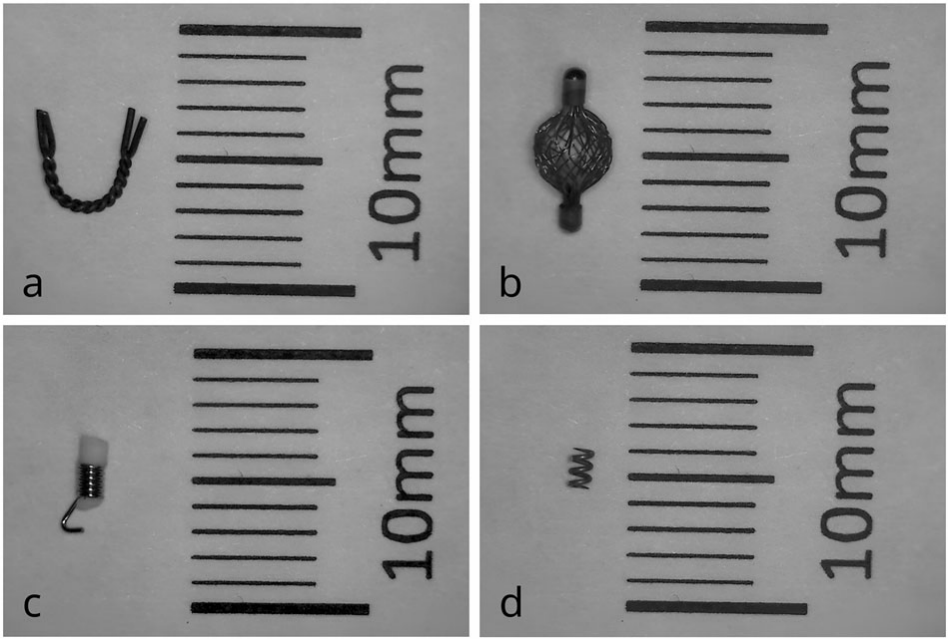
Photographs of the four biopsy clips: **a** TUMARK professional (Somatex-TP); **b** TUMARK Vision (Somatex-TV); **c** Bard-UC; **d** Devicor-HM

For each clip, an agar phantom with base diameters of 10 × 6 cm and a height of 15 cm was prepared (500 mL isotonic saline with 10 g of Agar Kobe I stirred in water heated to 90 °C), similar to Kato et al [14]. Clips used were Bard UltraClip Dual Trigger (Bard-UC), Somatex TUMARK professional (Somatex-TP), Somatex TUMARK vision (Somatex-TV), and devicor mammatome hydromark breast biopsy site marker (Devicor-HM) (Fig. 1).

### MRI protocol and clip marker placement

According to our clinical routine, experiments were performed on a 1.5-T and a 3-T whole-body scanner (Magnetom Avanto^Fit^ and Magnetom Skyra, Siemens Healthineers, Erlangen, Germany) using an 18-channel breast coil. Four different MRI conditional biopsy clips were investigated (Table 1 and Fig. 1).

**Table 1:** Investigated biopsy clips

Clip		Manufacturer product code	Manufacturer	Material	Shape	Length, (mm)
Somatex-TP	TUMARK professional	271560	Somatex Medical	Nitinol	U-shape	12.0
Somatex-TV	TUMARK vision	271590	Somatex Medical	Nitinol	Spherical	7.0
Bard-UC	BARD ultraclip dual trigger	864017DL	BD Medical	BioDur 108	Coil	10.0
Devicor-HM	HydroMARK breast biopsy site marker	4010-02-15-T3	Mammotome	Titanium	Open coil	2.5

The breast coil was loaded with two of the phantoms on the left and right sides. After an empty measurement, one clip per phantom was inserted at a depth of 60 mm using the placement system provided with the clips. Utmost care was taken not to cause any air inclusion.

Following current state-of-the-art recommendations [12, 15], the protocol consisted of the following sequences: T2-weighted fast spin-echo imaging with short inversion time fat-suppressed inversion-recovery [16] and magnitude reconstruction (T2-TIRM); T1-weighted fat-suppressed spoiled gradient-echo (T1_FL3D), which in our clinical routine is used for dynamic contrast-enhanced imaging; and DWI. For DWI two different sequences were used: a readout segmented echo-planar imaging (RESOLVE-DWI) sequence [17, 18] with the same parameters as used for patient exams at our institution: and an echo-planar imaging sequence (EPI-DWI) which is frequently used when RESOLVE-DWI is not available.

Imaging parameters for all sequences are given in Table 2. Imaging was started at least 24 h after clip placement to allow for the hydration of the HydroMARK Breast Biopsy Site Marker coating. Measurements at 1.5 T and 3 T were always performed on the same day.

**Table 2:** Sequence parameters

	T2-TIRM	T1_FL3D	RESOLVE-DWI	EPI-DWI
Repetition time, (ms)	5,150	5.32	8,710	4,900
Echo time, (ms)	61	1.93	61	68
Inversion time, (ms)	@1.5 T: 170 @ 3 T: 230	–	–	–
Flip angle, (°)	90	10	90	90
Turbo factor	11	–	5	108
Receive bandwidth, (Hz/Px)	228	300	610	1,984
Fat suppression	STIR	SPAIR	SPAIR	SPAIR
Parallel imaging	GRAPPA	GRAPPA	GRAPPA	GRAPPA
Acceleration factor	3	3	2	2
*b*-values, (s/mm^2^)	–	–	50/400/800	50/400/800
Averages	2	1	1/3/5	2/4/4
Slice thickness, (mm)	4	1.6	4	4
Number of slices	35	112	42	30
Slice orientation	Transversal	Transversal	Transversal	Transversal
Field of view, (mm *×* mm)	300 *×* 300	300 *×* 300	360 *×* 180	340 *×* 204
Acquisition matrix	448 *×* 336	480 *×* 422	216 *×* 98	180 *×* 108
Acquisition time, (min:s)	2:41	1:27	4:23	2:52

### Measurements

All images were analyzed using our institution’s picture archiving and communication system—PACS software Impax EE (R20 XVII SU1, Agfa, Mortsel, Belgium). The dimensions of the visible artifact were quantified on the image with the largest appearance of the artifact by tracing the outlines of the visible signal changes on this image.

**Fig. 2:**
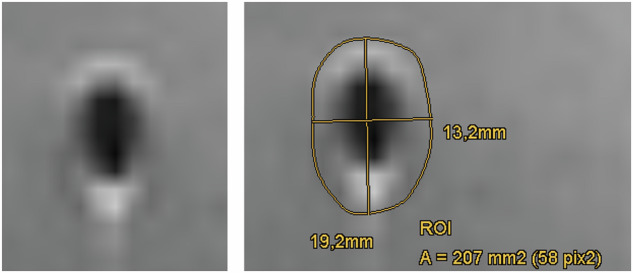
Illustration of artifact quantification on the slice with the largest appearance of the artifact by tracing the outlines of the visible signal changes. The area of the obtained spline-interpolated polygonal ROI and the long- and short-axis of this ROI were recorded

The area of the obtained spline-interpolated polygonal region of interest (ROI) and the extent of the long and short axis of this ROI were recorded (Fig. 2). For DWI, the extent of the visible artifact was evaluated on the diffusion-weighted images (*b* = 0, 400 s/mm^2^, or 800 s/mm^2^), as well as on the apparent diffusion coefficient (ADC) maps. In addition, the ADC maps were also inspected for ADC changes. For this purpose, ADC values were obtained from ADC maps after placing circular ROIs well outside the region of visible artifact on DWI images, within the region of visible artifact on the ADC maps, and within a region of the DWI artifact which was still outside of the visible artifact on the ADC maps. To assess repeatability MRI of the phantoms was repeated one month after the first experiment and a third time one week after the second imaging session. To assess interrater and interrater variability the quantification of the artifacts was repeated for one measurement time by the same reader (C.K., physicist with 30 years of MRI experience) and independently by a second reader (L.G., radiologist with 7 years of MRI experience).

### Statistical analysis

Measures of artifact size are given as median and range over the available data from all experiments and both readers. All statistical calculations were performed using the R Project for Statistical Computing 4.2.1 software [19]. Inter- and interrater variability was determined by calculating the intraclass correlation coefficient using the “*irr*” package for R [20]. For pairwise comparison of artifact sizes, such as between the different clips or between different DWI *b-va*lues, Analysis of variance (ANOVA) was performed using Tukey’s method for multiple comparisons. To compare differences in artifact size between 1.5 T and 3 T for individual clips and sequences, or between DWI and ADC a Wilcoxon rank sum test with continuity correction was used. Results were considered significant for *p*-values lower than 0.050.

## Results

The extent of the obtained artifacts is listed in Tables 3 and 4 for all clips, all applied sequences, and the two field strengths. Typical images of the artifacts are given as Supplementary Material. Table 5 shows the significance levels for the difference of artifact area between the different markers. Artifact size ranged from 5.7 × 8.5 mm^2^ to 13.4 *×* 17.7 mm^2^ (area between 40.3 mm^2^ and 188 mm^2^) at 1.5 T and 6.6 *×* 8.2 mm^2^ to 17.7 *×* 20.7 mm^2^ (area between 45.3 mm^2^ and 306 mm^2^) at 3 T. The interrater agreement for the obtained artifact dimensions was high, with an intraclass correlation coefficient for the artifact area (based on freehand ROIs, see Fig. 2) of 94.8% (95% confidence interval [CI]: 89.1–97.2%), for the transversal artifact diameters of 94.5% (95% CI: 87.1–97.2%) and for the longitudinal diameters of 90.7% (95% CI: 85–94.3%). The reproducibility of artifact sizes between repeated acquisitions of the same phantoms was between 70.0% and 91.4%. Inter-rater agreement for the artifact area (based on freehand ROIs, see Fig. 2) was 85.7% (95% CI: 69.9–92.5%), 90.6% (95% CI: 84.8–94.2%) for the longitudinal diameters and 80.4% (95% CI: 55.2–90.2%) for the transversal diameters.

**Table 3:** Extent of artifacts at 1.5 T

Clip	Long axis, (mm)	Transversal axis, (mm)	Number	Artifact area, (mm^2^)
T2-TIRM				
Somatex-TP	8.5 (7.9–8.9)	5.7 (4.8–7.0)	3	40.3 (35.9–46.2)
Somatex-TV	11.3 (10.7–11.4)	6.2 (5.3–6.9)	3	61.6 (47.9–67.9)
Bard-UC	7.9 (7.7–8.6)	7.6 (6.4–8.6)	3	50.2 (42.6–60.4)
Devicor-HM	11.3 (10.8–11.7)	6.9 (6.4–7.3)	2	68.1 (60.0–72.7)
T1_FL3D				
Somatex-TP	9.2 (8.7–10.1)	6.6 (6.0–7.1)	7	51.6 (44.4–60.6)
Somatex-TV	13.0 (11.1–13.3)	7.9 (7.6–8.9)	8	83.7 (71.5–93.6)
Bard-UC	11.2 (10.9–13.5)	11.0 (9.7–12.0)	11	98.6 (80.2–120.0)
Devicor-HM	5.4 (4.1–6.0)	4.7 (3.3–5.1)	6	22.3 (13.1–24.8)
RESOLVE-DWI				
Somatex-TP	13.4 (12.2–13.8)	9.3 (8.4–10.0)	5	96.6 (92.9–112.0)
Somatex-TV	16.3 (15.6–17.4)	9.7 (9.4–10.6)	4	121.0 (118.0–134.0)
Bard-UC	14.6 (13.6–15.2)	11.6 (11.4–13.8)	4	137.0 (124.0–155.0)
Devicor-HM	11.9 (10.6–12.0)	8.2 (7.8–9.9)	3	73.5 (72.0–97.9)
RESOLVEADC				
Somatex-TP	9.0 (6.1–12.0)	5.1 (3.4–10.0)	3	33.1 (16.7–92.2)
Somatex-TV	13.4 (8.5–15.8)	7.5 (6.9–9.1)	2	67.5 (47.1–114.0)
Bard-UC	8.5 (7.3–10.1)	7.0 (6.1–7.4)	2	49.5 (35.8–55.2)
Devicor-HM	9.6 (8.6–11.4)	6.9 (6.5–11.9)	2	53.6 (47.6–74.7)
EPI-DWI				
Somatex-TP	16.7 (16.2–17.0)	11.7 (9.6–13.6)	4	165.0 (138.0–181.0)
Somatex-TV	18.6 (16.8–19.4)	11.4 (10.8–13.2)	4	177.0 (154.0–207.0)
Bard-UC	17.7 (15.9–18.6)	13.4 (12.9–14.2)	4	188.0 (180.0–213.0)
Devicor-HM	13.2 (12.1–19.1)	9.4 (8.7–14.6)	2	145.0 (86.8–166.0)
EPI-ADC				
Somatex-TP	10.2 (10.1–13.5)	8.0 (6.9–10.0)	3	81.2 (56.3–93.3)
Somatex-TV	11.8 (10.4–13.5)	6.0 (5.3–6.5)	2	53.5 (50.6–70.6)
Bard-UC	11.0 (9.3–12.0)	8.4 (7.6–9.9)	2	75.0 (68.6–151.0)
Devicor-HM	9.0 (5.3–14.5)	7.1 (4.9–8.4)	2	59.7 (19.9–97.0)

Given values are median values overall measurements, with the range of values shown in parentheses. For DWI images, the *b-va*lue is not specified in the table, because no significant difference in artifact size between the acquired *b-va*lues (*b* = 50 s/mm^2^, 400 s/mm^2^, and 800 s/mm^2^) was found (*p* = 0.911–1.000 for 1.5-T and 3-T)

*Bard-UC* BARD ultraclip dual trigger, *Devicor-HM* Hydromark breast biopsy site marker, *n* the median number of slices where the artifact was visible, *Somatex-TP* TUMARK professional, *Somatex-TV* TUMARK vision

**Table 4:** Extent of artifacts at 3 T

Clip	Long axis, (mm)	Transversal axis, (mm)	Number	Artifact area, (mm^2^)
T2-TIRM				
Somatex-TP	8.2 (7.8–8.4)	6.6 (6.0–7.2)	3	45.3 (40.4–49.4)
Somatex-TV	11.0 (10.9–11.3)	6.6 (6.1–7.3)	3	61.6 (57.9–66.8)
Bard-UC	9.4 (8.7–9.5)	8.7 (8.5–8.8)	4	67.1 (61.8–69.4)
Devicor-HM	10.4 (9.0–11.1)	6.4 (4.6–7.1)	3	56.8 (38.5–68.5)
T1_FL3D				
Somatex-TP	9.6 (9.3–9.9)	7.3 (6.4–7.7)	11	56.7 (52.9–60.3)
Somatex-TV	12.9 (11.6–13.4)	7.9 (7.5–8.3)	10	81.4 (77.6–92.2)
Bard-UC	11.0 (10.0 – 12.1)	10.8 (9.9–11.9)	13	97.0 (84.3–113.0)
Devicor-HM	6.5 (5.5–9.8)	5.6 (5.3–7.0)	7	31.4 (25.2–58.2)
RESOLVE-DWI				
Somatex-TP	14.4 (14.1–15.4)	8.7 (7.8–10.3)	4	109.0 (92.2–121)
Somatex-TV	18.1 (16.4–18.8)	9.8 (9.0–11.7)	4	142.0 (134.0–174.0)
Bard-UC	15.6 (15.2–17.8)	12.8 (12.2–13.7)	6	159.0 (153.0–213.0)
Devicor-HM	11.8 (10.5–12.2)	8.5 (7.4–9.0)	3	73.0 (72.2–88.0)
RESOLVE-ADC				
Somatex-TP	12.6 (11.9–14.4)	8.1 (5.4–8.8)	3	81.6 (52.3–95.0)
Somatex-TV	13.0 (12.9–17.6)	7.2 (5.4–9.4)	2	85.4 (52.4–131.0)
Bard-UC	14.2 (12.6–14.8)	11.0 (8.7–11.3)	4	128.0 (97.1–138.0)
Devicor-HM	14.2 (11.0–17.0)	6.1 (5.8–6.5)	2	72.5 (52.7–95.4)
EPI-DWI				
Somatex-TP	18.5 (17.9–19.7)	11.8 (11.0–14.4)	4	176.0 (160.0–223.0)
Somatex-TV	21.5 (20.5–25.3)	13.6 (12.0–14.7)	4	227.0 (212.0–276.0)
Bard-UC	20.7 (19.9–22.9)	17.7 (14.6–17.9)	5	306.0 (227.0–334.0)
Devicor-HM	15.2 (13.3–23.6)	10.1 (8.4–11.4)	3	131.0 (88.6–174.0)
EPI-ADC				
Somatex-TP	13.1 (10.6–18.0)	7.6 (7.0–11.6)	3	77.4 (65.1–156.0)
Somatex-TV	15.8 (12.1–17.8)	7.5 (6.3–12.3)	2	94.1 (80.8–187.0)
Bard-UC	13.8 (9.7–14.2)	10.2 (8.6–12.9)	3	117.0 (68.6–151.0)
Devicor-HM	8.5 (7.5–18.1)	5.4 (4.5–6.0)	1	35.9 (34.9–82.3)

Given values are median values overall measurements, with the range of values shown in parentheses. For DWI images, the *b-va*lue is not specified in the table, because no significant difference in artifact size between the *b-va*lues (*b* = 50 s/mm^2^, 400 s/mm^2^, and 800 s/mm^2^) (*p* = 0.911–1.000 for 1.5-T and 3-T)

*Bard-UC* BARD ultraclip dual trigger, *Devicor-HM* Hydromark breast biopsy site marker, *n* the median number of slices where the artifact was visible, *Somatex-TP* TUMARK professional, Somatex-TV TUMARK vision

**Table 5:** Values of *p* for the differences in artifact area between clips

*B*_0_ = 1.5 T						
	Devicor-HM *versus* Bard-UC	Somatex-TP *versus* Bard-UC	Somatex-TV *versus* Bard-UC	Somatex-TP *versus* Devicor-HM	Somatex-TV *versus* Devicor-HM	Somatex-TV *versus* Somatex-TP
T2-TIRM	0.004	0.056	0.264	< 0.001	0.172	0.001
T1_FL3D	< 0.001	< 0.001	0.092	< 0.001	< 0.001	< 0.001
RESOLVE-DWI	< 0.001	< 0.001	0.188	0.011	< 0.001	0.011
RESOLVE-ADC	0.830	0.988	0.305	0.951	0.773	0.467
EPI-DWI	0.002	0.159	0.751	0.133	0.013	0.622
EPI-ADC	0.504	0.999	0.622	0.424	0.997	0.537

*B*_0_ = 3 T						
	Devicor-HM *versus* Bard-UC	Somatex-TP *versus* Bard-UC	Somatex-TV *versus* Bard-UC	Somatex-TP *versus* Devicor-HM	Somatex-TV *versus* Devicor-HM	Somatex-TV *versus* Somatex-TP
T2-TIRM	0.104	0.002	0.868	0.227	0.356	0.009
T1_FL3D	< 0.001	< 0.001	0.183	0.060	< 0.001	0.005
RESOLVE-DWI	< 0.001	< 0.001	0.096	0.035	< 0.001	0.009
RESOLVE-ADC	0.007	0.016	0.107	0.981	0.531	0.753
EPI-DWI	< 0.001	< 0.001	0.090	0.095	< 0.001	0.092
EPI-ADC	0.053	0.797	0.994	0.261	0.032	0.652

For the absolute values see Tables 3 and 4

Independently of field strength, the following observations were made (see Tables 3 and 4). At both field strengths, all clips showed the largest artifact area on EPI-DWI followed by Resolve-DWI (*p* ≤ 0.016). T2-TIRM showed the smallest artifact area for Somatex-TP, Somatex-TV, and Bard-UC (*p* ≤ 0.002), while for Devicor-HM the artifact was smallest on T1_FL3D (*p* = 0.026; see Supplementary Data, Figs. S5 and S6). On T1_FL3D, RESOLVE-DWI, and EPI-DWI images, the Bard-UC clip generated the largest artifact, followed by the Somatex-TV and Somatex-TP clips, while the smallest artifact was observed for the Devicor-HM clip. For all sequences, there was no significant difference between Somatex-TV and Bard-UC. Interestingly, on T2-TIRM images, the smallest artifact was seen for the Somatex-TP clip. No significant difference in artifact size was observed between DWI images for different *b-va*lues (*b* = 50 s/mm^2^, 400 s/mm^2^, or 800 s/mm^2^) (*p* ≥ 0.911 for all cases).

Regarding the T1_FL3D sequence, although the overall mean artifact area was lower for Devicor-HM than for the Somatex-TP clip, the difference was only significant at 1.5 T (*p* < 0.001), not at 3 T (*p* = 0.060). Similarly, for the T2-TIRM sequence, the Somatex-TP showed an overall lower artifact area than the Devicor-HM clip, the difference in size being significant at 1.5 T (*p* < 0.001), not at 3 T (*p* = 0.227).

On T2-TIRM and DWI images, the artifacts presented as a signal void and a bright rim for all clips except for the Devicor-HM, which showed a marked signal increase and a dark rim (see Supplementary Material, Figs. S1, S3, and S4). For the T1_FL3D sequence, a signal void was observed for all markers. Outside of the visible artifact, no additional image degradation could be observed.

**Fig. 3:**
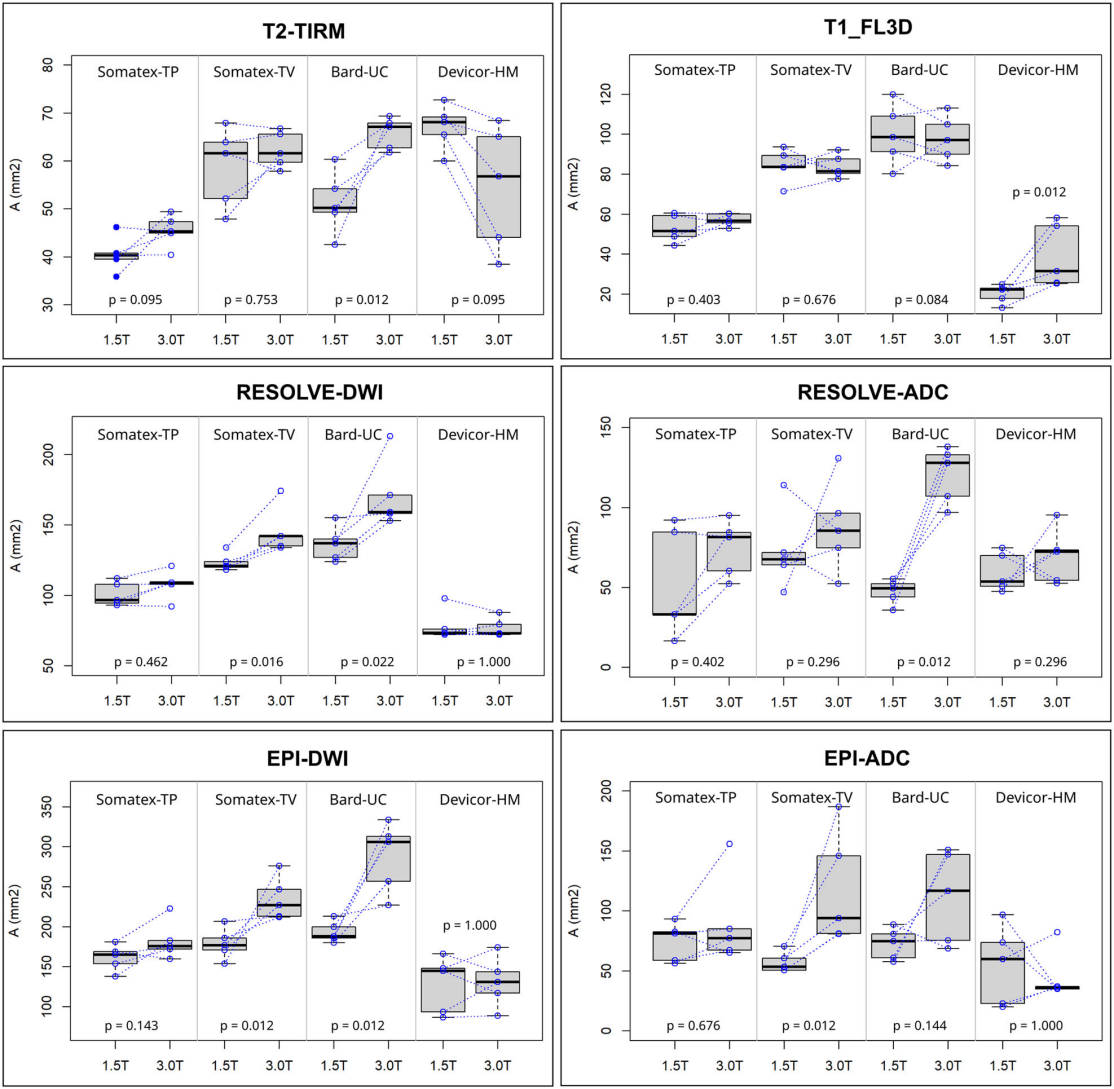
Data of all measurements for each clip and field strength were pooled. Measurements at 1.5 T and 3 T were always performed on the same day. The results of these individual measurements are indicated as blue circles. Same-day measurements are connected by dotted lines

Figure 3 shows the change of the artifact area for the different clips and different sequences between 1.5 T and 3 T. Somatex-TP did not show a significant change of artifact area for all sequences. Somatex-TV revealed a significant change of artifact area only for RESOLVE-DWI, EPI-DWI, and EPI-ADC (*p* = 0.016, *p* = 0.012, and *p* = 0.012, respectively) There was a significant increase of artifact area observed for Bard-UC on T2-TIRM (*p* = 0.012), RESOLVE-DWI (*p* = 0.022), RESOLVE-ADC (*p* = 0.012), and EPI-DWI (*p* = 0.012), but not on T1_FL3D (*p* = 0.835) and EPI-ADC (*p* = 0.144). Interestingly, for Devicor-HM, a significant increase in artifact size was observed for T1_FL3D (*p* = 0.012), but not for the other sequences.

**Fig. 4:**
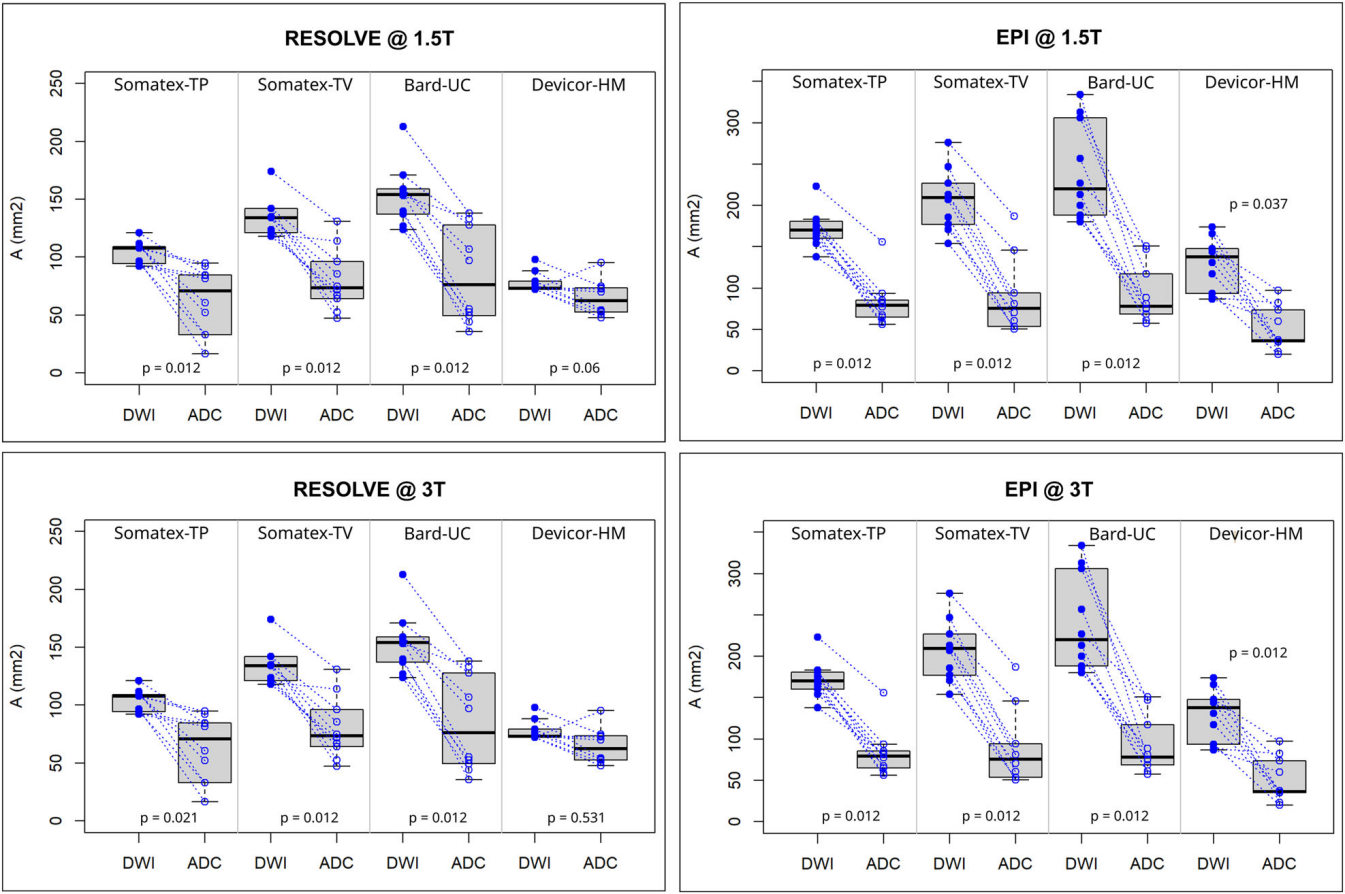
Comparison of artifact area between diffusion-weighted images (DWI) and ADC maps for the different clips. For the shown boxplots, data of all measurements for each clip and field strength were pooled. Measurements at 1.5 T and 3 T were always performed on the same day. The results of these individual measurements are indicated as blue circles. Same-day measurements are connected by dotted lines

Finally, as shown in Fig. 4, a significant decrease in artifact area for all clips from DWI to ADC images was found, with the exception of Devicor-HM for the RESOLVE sequence (see Supplementary Data, Fig. S3). Comparing ADC values well outside the DWI artifact and the region within the DWI artifact but outside the artifact on the ADC map for all clips did not show a significant difference (*p* = 0.622). Significant differences in ADC values were only observed inside the artifact visible on the ADC maps.

## Discussion

In this *in vitro* study, we evaluated and compared MRI artifacts produced by four different types of breast clip markers commonly used for marking biopsy sites of breast lesions, for various sequences at 1.5 T and 3 T field strengths. The sequences chosen for this study encompass those considered standard for assessment and local staging [13].

Of note, the selection of a breast biopsy marker is frequently guided by the visibility of the marker at different imaging modalities such as ultrasound or mammography, which is governed by the individual characteristics of the marker [21]. Marker clips can be either ‘bare’, as preferred for small or superficial lesions, or combined with a bioabsorbable material to enhance ultrasound visibility, improve hemostasis, or reduce migration [9, 21]. Whereas the clips TUMARK professional, TUMARK vision, and BARD-UC, used in our study, belong to the ’bare’ category, the Devicor-HM clip is coated with a bioabsorbable hydrogel, which extents within 24 h after deployment by absorbing water and provides significantly increased ultrasound visibility for 12–15 months [21]. In contrast, to avoid protrusion through the skin, ‘bare’ markers should be favored for superficial lesions. In addition, ‘bare’ markers are also chosen for small lesions if precise spatial marking of a given lesion is necessary or to reduce the risk of allergy to nickel found in some other clips, even though considered a very rare complication [22].

While breast MRI is the follow-up mainstay for screening high-risk patients due to familial history and/or known gene defects and a breast cancer lifetime risk greater than 20% [12, 23], over recent years, breast MRI has been suggested as a primary local staging tool for select patients with newly diagnosed breast cancer [12] and in cases with unclear findings at mammography or ultrasound, where a biopsy does not seem warranted [24, 25].

We found that the size, shape, and appearance of artifacts on MRI depend on the design of the device and vary among different clips and MRI sequences. The artifact size ranged from 5.7 × 8.5 mm^2^ to 13.4 *×* 17.7 mm^2^ at 1.5 T and from 6.6 *×* 8.2 mm^2^ to 17.7 *×* 20.7 mm^2^ at 3 T. These findings are in line with a recent study by Puesken et al [25], who reported for ten different commercially available clip markers a similar range of artifact sizes and indicated that clip artifacts can occupy a considerable portion of the image, potentially affecting the visibility of adjacent breast tissue and other structures. The author followed an *in vitro* approach, not assessing diffusion-weighted sequences.

The high interrater agreement for artifact size suggests consistent and reliable measurements of the artifact among the observers. Similarly, the reproducibility of artifact size between repeated acquisitions of the same phantoms indicates the stability of the clip artifacts under similar imaging conditions. The interrater agreement for artifact size was slightly lower than the interrater agreement, indicating some variability in the perception of artifact size among different observers. However, the agreement was still relatively high, suggesting that the clip artifacts were consistently discernible across different raters. It is worth noting that interrater agreement for the longitudinal extent of the artifact was slightly lower than for the artifact area and transversal extent. This could be due to differences in subjective interpretation of the boundaries of the artifact along the longitudinal direction.

The comparison of artifact size among the different clips and MRI sequences revealed notable findings. On T1_FL3D, RESOLVE-DWI, and EPI-DWI sequences, the Bard-UC clip consistently produced the largest artifact, followed by Somatex-TV and Somatex-TP, while the smallest artifact was observed for Devicor-HM. This suggests that the Bard-UC clip may cause more pronounced image distortions and signal voids compared to the other clips, potentially compromising the visibility of nearby anatomical structures [11, 25]. Notably, no significant difference in artifact size was observed between Somatex-TV and Bard-UC for all field strengths and sequences, indicating that these two clips had similar effects on local image quality.

On T2-TIRM images, Somatex-TP consistently showed the smallest artifact size at both field strengths, while Devicor-HM exhibited a larger artifact size, most likely caused by its hydrogel-coating [26, 27]. These findings suggest that Somatex-TP may cause fewer signal voids and distortions on T2-weighted images and that the Devicor-HM may negatively affect local assessment where T2-weighted imaging is essential for lesion characterization. There was a significant difference in artifact size between Devicor-HM and Somatex-TP at 1.5 T but with *p* = 0.060, the difference was inconclusive at 3 T. The low certainty of the difference at 3 T is due to the low number of samples and the observed high variability of the area measurements for Devicor-HM at 3 T, probably due to the mentioned hydrogel-coating. This also indicates that the impact of the clips on local image quality can be field strength-dependent. It is important for radiologists to be aware of these variations when interpreting MRI scans with different field strengths. Artifacts may thus potentially completely obscure lesions, making a proper measurement and tissue characterization impossible, which is especially noteworthy in lesions occult in other modalities [12].

Even with the Devicor-HM clip, which produced the smallest artifact for all investigated clips, for the used sequence parameters, in some cases, lesions up to 10 mm in maximum diameter might be obscured. This may have less relevance in clinical practice, as clip markers may not always be found strictly centrally within small lesions due to initial placement, migration, or dislocation.

Interestingly, no significant difference in artifact size was observed between DWI images for the different acquired *b-va*lues at both 1.5 T and 3 T. This suggests that the clip artifacts may not be significantly influenced by the diffusion-weighting factors in the DWI sequences used in this study. The consistent appearance of the clip artifacts as signal voids and bright rims for all clips except Devicor-HM indicates a consistent pattern of signal alteration caused by the clips. In contrast, Devicor-HM showed a marked signal increase and a dark rim, most likely relying on the hydrogel-coating [26, 27]. These findings highlight the importance of understanding the unique characteristics of each clip type and their impact on image interpretation.

The clinical implications of clip artifacts, particularly for local staging of small cancers, are noteworthy. In local staging, accurate assessment of tumor size and proximity to surrounding structures is crucial for treatment planning and determining surgical margins. The presence of clip artifacts can potentially obscure or distort the tumor margins, making it challenging to accurately measure tumor size and assess its relationship with adjacent structures [11]. Furthermore, significant artifact superimposition may hamper the assessment of a tumor’s response to neoadjuvant therapy, as tumors may regress beyond the artifacts border. Here, additional evaluation by ultrasound or contrast-enhanced mammography may be necessary.

**Fig. 5:**
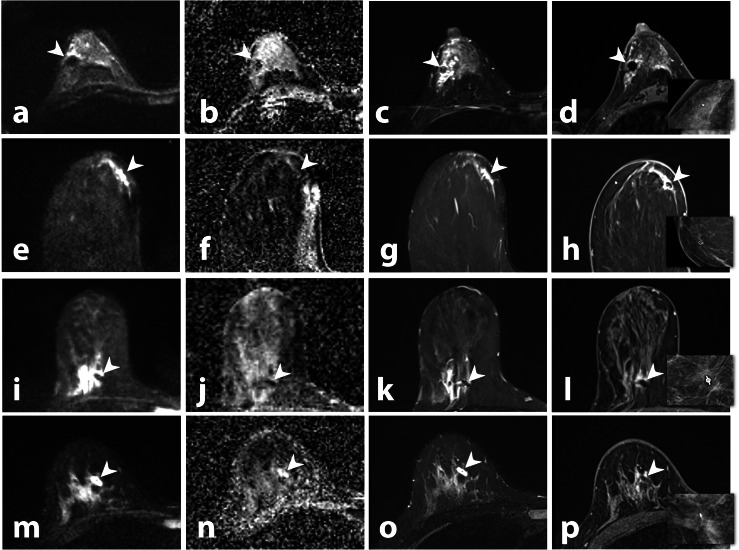
Clip artifact examples (white arrowheads) on diffusion-weighted images (*b* = 800 s/mm^2^) (1st column); ADC maps (2nd column). T2-weighted turbo inversion-recovery magnitude images (3rd column), and contrast-enhanced fast-suppressed T1_Flash3D images (4th column) for Bard-UC (**a**–**d**), Somatex-TP (**e**–**h**), Somatex-TV (**i**–**l**), and devicor mammatome hydromark (**m**–**p**). Small inserts depict mammographic post-biopsy clip control findings (lower right angle in the 4th column)

Accordingly, radiologists and clinicians should be aware of the extent and characteristics of clip artifacts produced by different clip types to mitigate the potential impact on local staging and treatment response assessment. Typical patient examples for the different clips are shown in Fig. 5.

Our study has limitations. The impact of clip artifacts on contrast enhancement could not be assessed in this study. Complementary *in vivo* studies should evaluate the artifact's impact on the visibility of tumors regarding contrast enhancement and ADC behavior. Due to the lack of inherent architectural features of the agarose-gel phantom, local image distortion beyond the visibly discernible artifacts could not be assessed. In contrast to Puesken et al [25], we considered the T2-hyperintense rim caused by the Devicor-HM hydrogel-coating as part of the overall artifact extent, as this coating may also displace tissue and alter its MRI properties. Additionally, the Devicor-HM clip gradually decreases in water content and volume over time [21] and may show a shrinking artifact extent. The used phantom was made of homogeneous water-based agar and did not include dummy lesions so that the minimum size of the detectable lesion could only be deduced from the extent of the visible signal void including a possible hyperintense rim. However, because the artifact is due to magnetic field inhomogeneities introduced by the implanted clip, its size should not depend on the existence of eventual dummy lesions within the phantom. Nevertheless, the exact size of the artifact might depend on sequence parameters like echo time, receive bandwidth, or fat suppression, so that, strictly speaking, the given minimum size of detectable lesion only applies to the sequence parameters used in the study. It should also be noted that the appearance of the artifact in patient-based measurements might vary compared to our phantom measurements due to different *in situ* orientations of the clips and due to an inhomogeneous environment containing lesions, fat, and blood vessels.

In conclusion, this *in vitro* study evaluated and compared the clip artifacts produced by four commonly used breast clip markers using various MRI sequences at 1.5 T and 3 T field strengths. We demonstrate considerable variations in artifact size and characteristics among the different clips and sequences. No significant difference in artifact size was observed between DWI images for various *b-va*lues. For all investigated clips and sequence parameters, a considerable part of certain smaller lesions might be obscured by clip artifacts, depending on the sequence and clip type. The clinical implications of clip artifacts for local staging in smaller carcinomas include potential challenges in accurately assessing tumor size and its relationship with surrounding structures. Awareness of these artifacts and their characteristics is essential for radiologists and clinicians to ensure accurate interpretation of MRI scans and appropriate treatment planning. Further, *in vivo* studies are warranted to investigate the impact of clip artifacts on clinical decision-making and patient outcomes.

## Supplementary information

**Additional file 1:** **Supplementary Fig. S1:** Typical images for t2-term showing the slice with the largest appearance of the artifact for all markers at 1.5 T and 3 T. Artifacts presented as a signal void and a bright rim for all clips except for HM, which showed a marked signal increase and a dark rim. **Supplementary Fig. S2:** Typical images for t1_fl3d showing the slice with the largest appearance of the artifact for all markers at 1.5 T and 3 T. For the t1_fl3d sequence, a signal void was observed for all markers. **Supplementary Fig. S3:** Typical images for Resolve-DWI (**a**) and Resolve-ADC (**b**) showing the slice with the largest appearance of the artifact for all markers at 1.5 T and 3 T. On Resolve-DWI images artifacts presented as a signal void and a bright rim for all. A significant decrease of artifact area for all clips from DWI to ADC images was found. **Supplementary Fig. S4:** Typical images for EPI-DWI (**a**) and EPI-ADC (**b**) showing the slice with the largest appearance of the artifact for all markers at 1.5 T and 3 T. On EPI-DWI images artifacts presented as a signal void and a bright rim for all. A significant decrease of artifact area for all clips from DWI to ADC images was found. **Supplementary Fig. S5:** Comparison of artifact area between the investigated sequences for the different clips at 1.5 T. **Supplementary Fig. S6:** Comparison of artifact area between the investigated sequences for the different clips at 3 T.

## Data Availability

The datasets used and/or analyzed during the current study are available from the corresponding author upon reasonable request.

## Abbreviations

ADC: Apparent diffusion coefficient
Bard-UC: Bard ultraclip dual trigger
CI: Confidence interval
Devicor-HM: Devicor mammatome hydromark breast biopsy site marker
DWI: Diffusion-weighted imaging
EPI: Echo-planar imaging
MRI: Magnetic resonance imaging
RESOLVE: Readout segmented echo-planar imaging
Somatex-TP: Somatex TUMARK professional
Somatex-TV: Somatex TUMARK vision
T1_FL3D: T1-weighted fat-suppressed spoiled gradient-echo
T2-TIRM: T2-weighted fast spin-echo short inversion time fat-suppressed inversion-recovery with magnitude reconstruction
