# Supplementary material for: Quantification of breast biopsy clip marker artifact on routine breast MRI sequences: a phantom study
Source: Eur Radiol Exp. 2024 Nov 15;8:128. doi: 10.1186/s41747-024-00525-2 (PMC11568078; doi:10.1186/s41747-024-00525-2)
Supplement: Supplementary file 1 — Additional file 1: Supplementary Fig. S1: Typical images for t2-term showing the slice with the largest appearance of the artifact for all markers at 1.5 T and 3 T. Artifacts presented as a signal void and a bright rim for all clips except for HM, which showed a marked signal increase and a dark rim. Supplementary Fig. S2: Typical images for t1_fl3d showing the slice with the largest appearance of the artifact for all markers at 1.5 T and 3 T. For the t1_fl3d sequence, a signal void was observed for all markers. Supplementary Fig. S3: Typical images for Resolve-DWI (a) and Resolve-ADC (b) showing the slice with the largest appearance of the artifact for all markers at 1.5 T and 3 T. On Resolve-DWI images artifacts presented as a signal void and a bright rim for all. A significant decrease of artifact area for all clips from DWI to ADC images was found. Supplementary Fig. S4: Typical images for EPI-DWI (a) and EPI-ADC (b) showing the slice with the largest appearance of the artifact for all markers at 1.5 T and 3 T. On EPI-DWI images artifacts presented as a signal void and a bright rim for all. A significant decrease of artifact area for all clips from DWI to ADC images was found. Supplementary Fig. S5: Comparison of artifact area between the investigated sequences for the different clips at 1.5 T. Supplementary Fig. S6: Comparison of artifact area between the investigated sequences for the different clips at 3 T. [file 41747_2024_525_MOESM1_ESM.pdf]

Quantification of breast biopsy clip marker artifact on routine breast MRI sequences: a phantom study

ELECTRONIC SUPPLEMENTARY MATERIAL

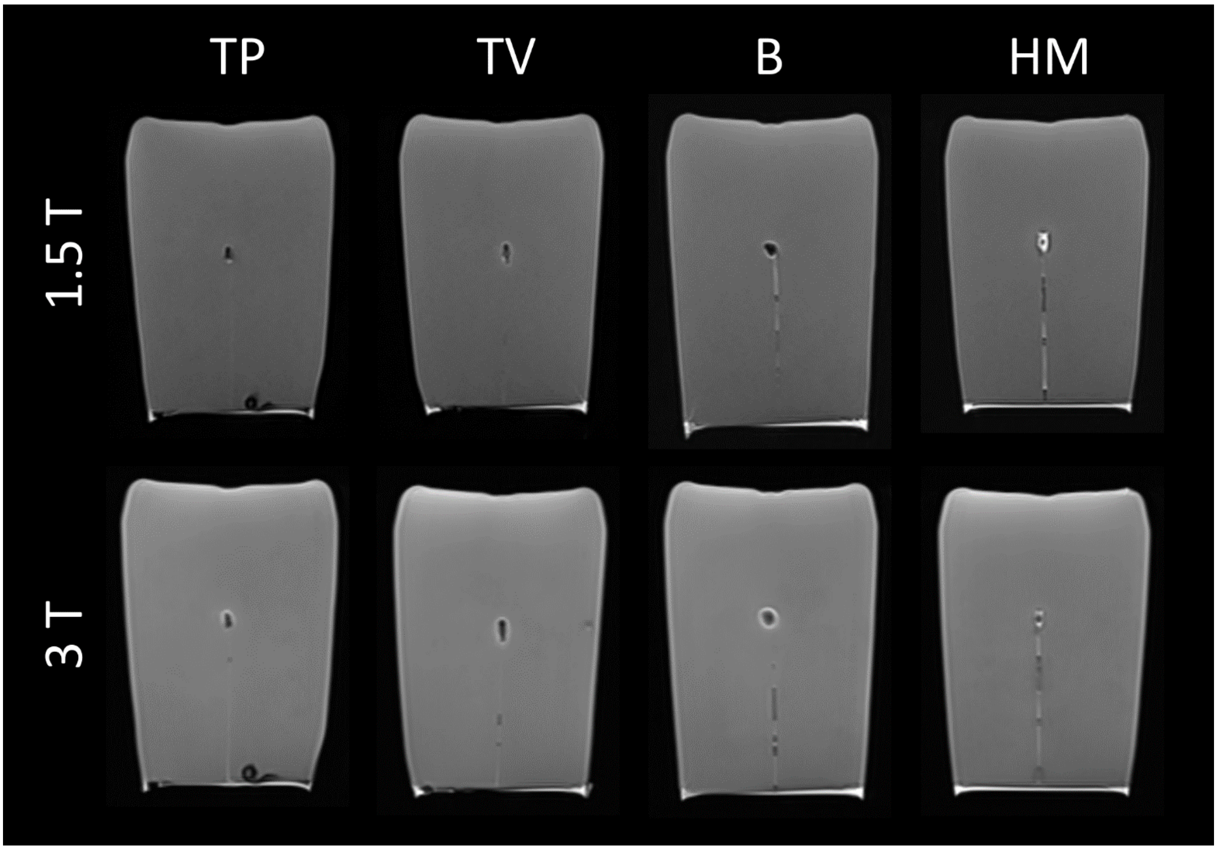

Supplementary figure S1:
Typical images for t2-tirm showing the slice with the largest appearance of the artifact for all markers at 1.5T and 3T. Artifacts presented as a signal void and a bright rim for all clips except for HM, which showed a marked signal increase and a dark rim.

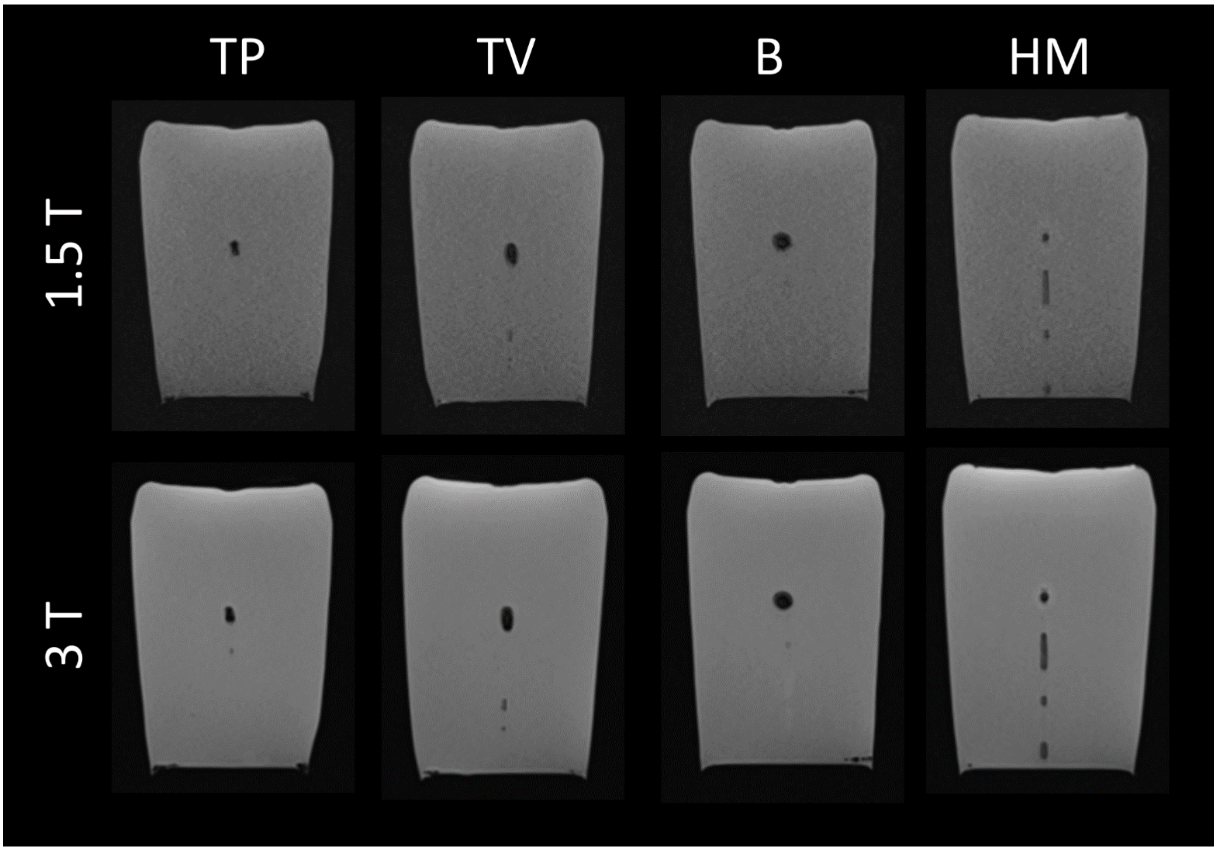

Supplementary figure S2:

Typical images for t1_fl3d showing the slice with the largest appearance of the artifact for all markers at 1.5T and 3T. For the t1_fl3d sequence, a signal void was observed for all markers.

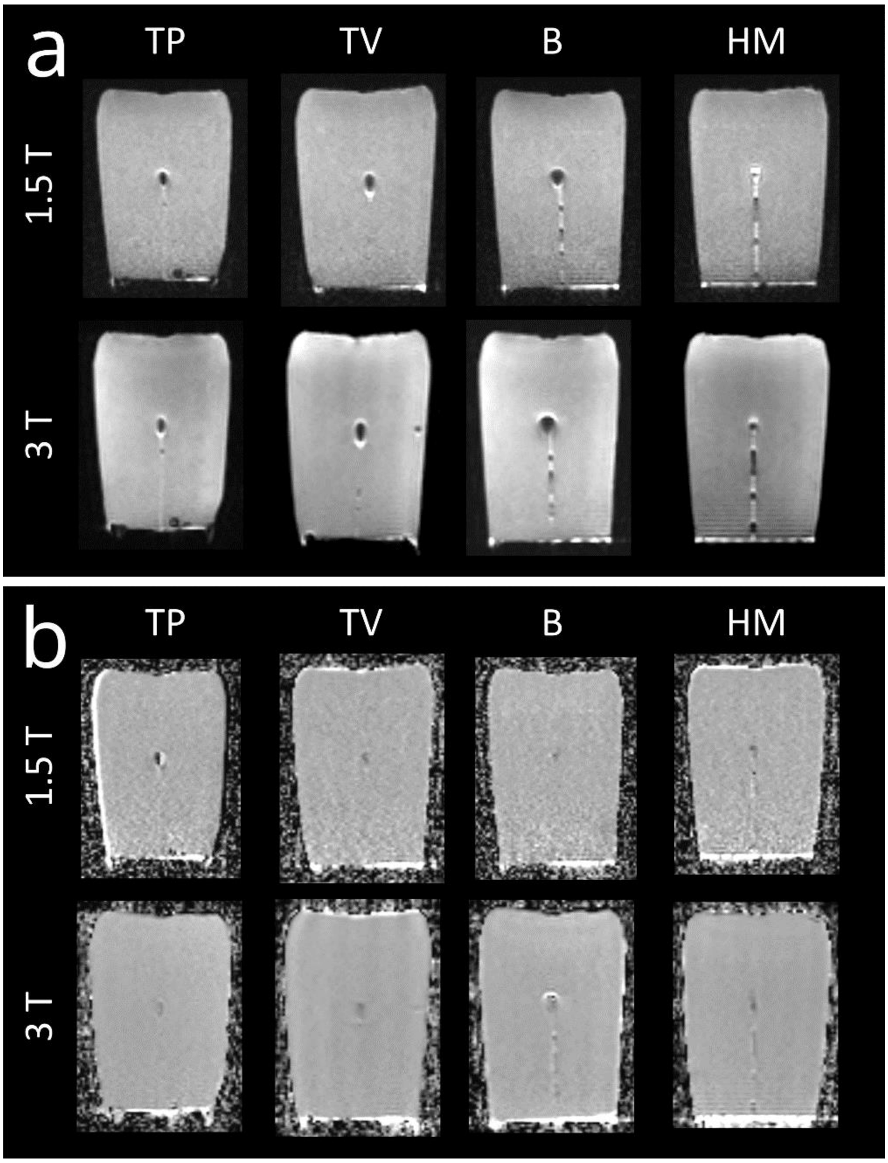

Supplementary figure S3:
Typical images for Resolve-DWI (a) and Resolve-ADC (b) showing the slice with the largest appearance of the artifact for all markers at 1.5T and 3T. On Resolve-DWI images artifacts presented as a signal void and a bright rim for all. A significant decrease of artifact area for all clips from DWI to ADC images was found.

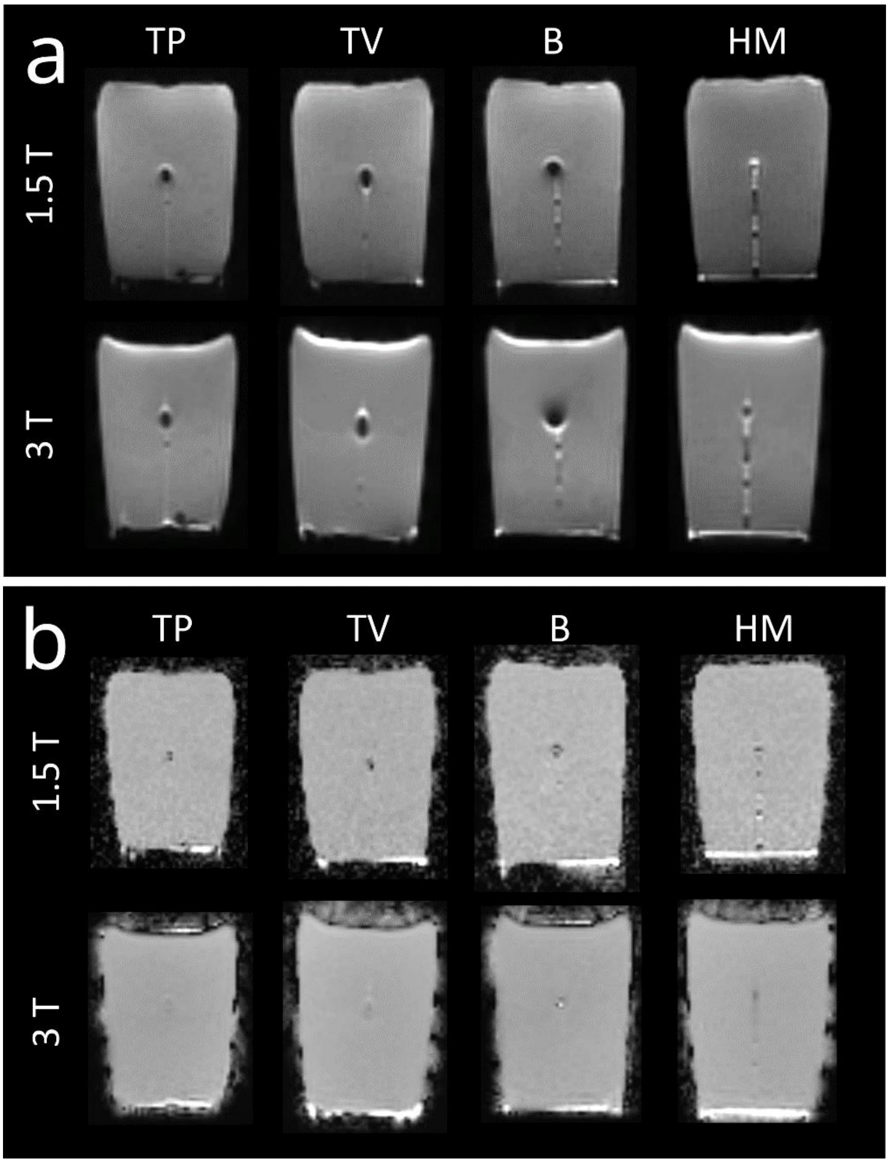

Supplementary figure S4:

Typical images for EPI-DWI (a) and EPI-ADC (b) showing the slice with the largest appearance of the artifact for all markers at 1.5T and 3T. On EPI-DWI images artifacts presented as a signal void and a bright rim for all. A significant decrease of artifact area for all clips from DWI to ADC images was found.

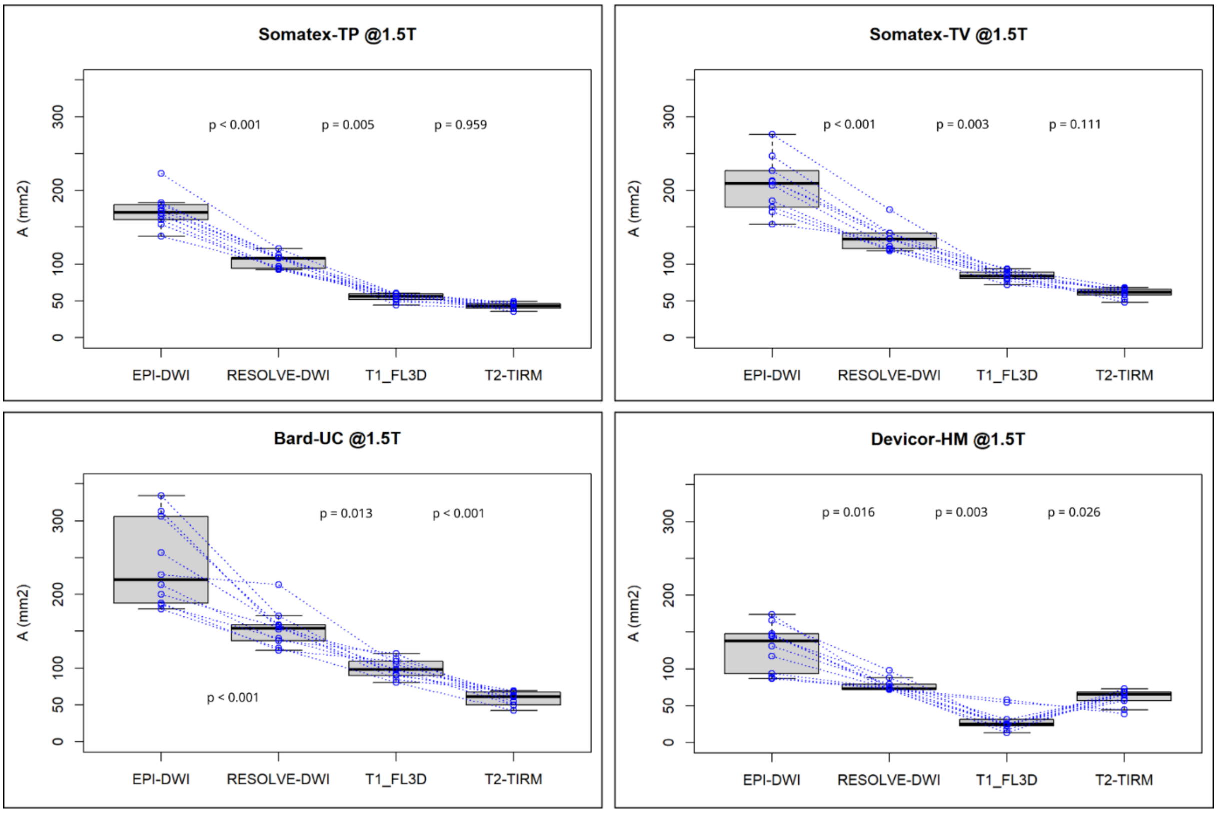

Supplementary figure S5:

Comparison of artifact area between the investigated sequences for the different clips at 1.5 T

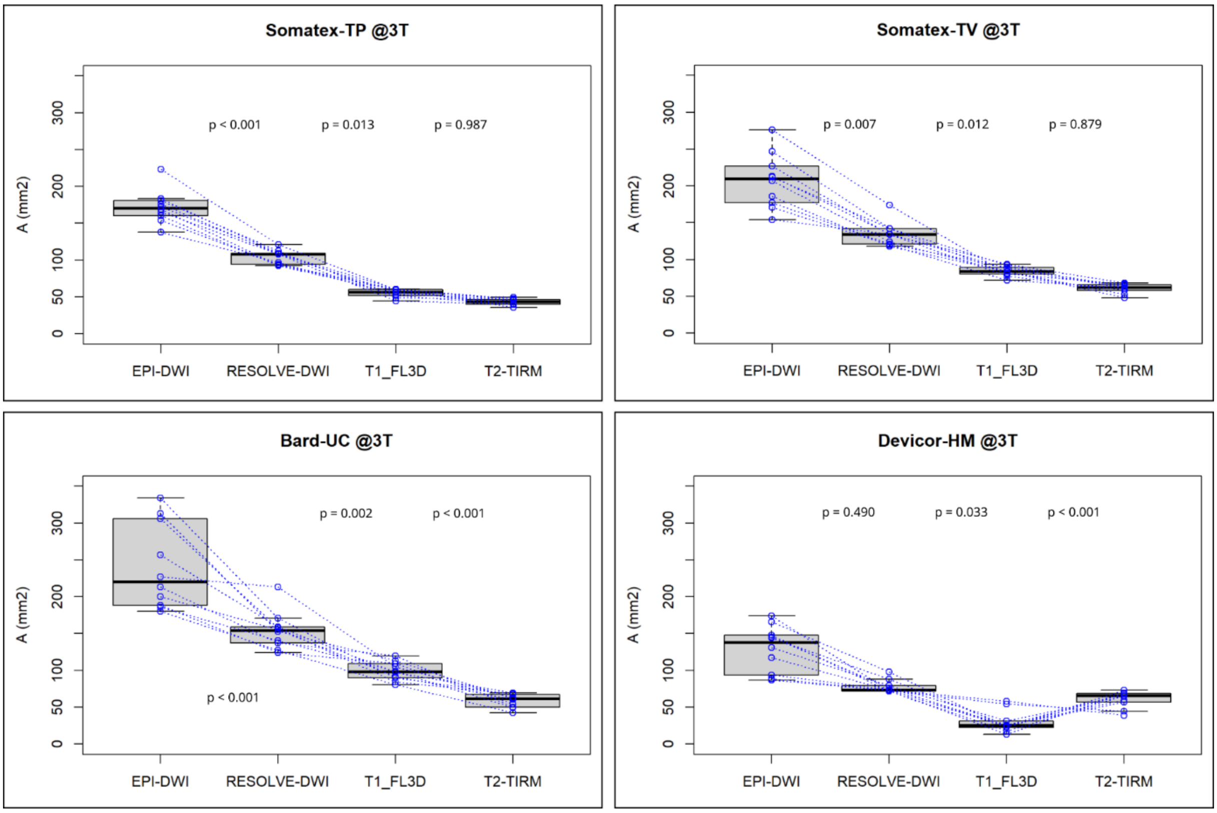

Supplementary figure S6:

Comparison of artifact area between the investigated sequences for the different clips at 3 T

Eur Radiol Exp (2024) Kremser C, Gruber L, Dietzel M, Amort B, Santner W, Daniaux M.
